# Feasibility of an app-based parent-mediated speech production intervention for minimally verbal autistic children: development and pilot testing of a new intervention

**DOI:** 10.1186/s40814-020-00726-7

**Published:** 2020-11-25

**Authors:** Jo Saul, Courtenay Norbury

**Affiliations:** 1 University College London Faculty of Brain Sciences, London, UK; 2 Department of Special Needs Education, University of Oslo, Oslo, Norway

## Abstract

**Background:**

Training speech production skills may be a valid intervention target for minimally verbal autistic children. Intervention studies have explored various approaches albeit on a small scale and with limited experimental control or power. We therefore designed a novel app-based parent-mediated intervention based on insights from the video modelling and cued articulation literature and tested its acceptability and usage.

**Methods:**

Consultation with the autism community refined the initial design and culminated in a pilot trial (*n* = 19) lasting 16 weeks. Participants were randomly allocated an intervention duration in an AB phase design and undertook weekly probes during baseline and intervention via the app. We evaluated the acceptability of the intervention via feedback questionnaires and examined the usability variables such as adherence to the testing and intervention schedule, time spent on the app and trials completed during the intervention phase.

**Results:**

High acceptability scores indicated that families liked the overall goals and features of the app. Ten participants engaged meaningfully with the app, completing 82% of the test trials and uploading data in 61% of intervention weeks; however, of these, only three met the targeted 12.5 min of intervention per week.

**Conclusion:**

We discuss the possible reasons for variability in usage data and how barriers to participation could be surmounted in the future development of this intervention.

**Supplementary Information:**

The online version contains supplementary material available at 10.1186/s40814-020-00726-7.

## Background

Multiple risk factors interact and combine to impact language acquisition in autism, and expressive language trajectories and outcomes are highly variable for autistic individuals. Approximately 25% of autistic individuals remain minimally verbal [1, 2], which means they have very limited ‘useful’ speech (i.e. speech used in frequent, communicative, non-imitative and referential ways [3]).

Development of functional speech by age 5 is one of the strongest predictors of positive adaptive outcome in adulthood [4], which has important implications for access to opportunities in the community, quality of life and independence. Identifying barriers to spoken language development and tailoring interventions accordingly are thus an important clinical and research aim.

Longitudinal studies have shown a host of variables to predict expressive language in young preverbal autistic cohorts (e.g. parent responsiveness, child joint attention skills and communicative intent), and these findings have informed intervention design (e.g. [5–7]). These studies have shown that parent and child interactive behaviour may be malleable, but downstream effects of enhanced joint engagement on child language measures are not always apparent.

Mounting evidence points to additional speech-motor barriers to language development in some autistic children [8, 9] which could explain different predictive patterns when older or more impaired cohorts are examined [10–12]. Consonant inventory is a commonly used measure of speech skills, which describes the number of different key consonants produced by the child in a language sample. Consonant inventory has been identified as an important predictor of expressive language growth in minimally verbal autistic children [12, 13]. The presence of motor-speech difficulties may call for a specific type of language intervention (e.g. in comparison with interventions where development of joint attention is presumed to be the underlying driver of expressive language growth).

The evidence base for interventions focusing on speech skills for minimally verbal autistic children is sparse. A systematic review of communication interventions for minimally verbal autistic children only identified two high-quality studies [14]. Only one of these targeted spoken language, and this was via a parent-mediated focused play therapy for 32–82-month-olds [15]. This intervention focussed on improving engagement and broad communication goals rather than speech skills. Approaches to improving speech production skills directly have mainly been evaluated by case series or small group studies, with limited power and experimental control over confounds. The majority of these used behavioural approaches such as discrete trial training [16–18], naturalistic child-led programmes [19] or combinations of these with Alternative and Augmentative Communication aids [20–22]. Non-behavioural approaches have included music- and/or rhythm-based techniques such as auditory-motor mapping training [23–25] or melodic-based communication therapy [26] and sensory-motor training [27, 28]. It is difficult to draw conclusions about the efficacy of these interventions, given the lack of robust well-powered evaluations to date. However, common themes include (1) improvements in target behaviours (e.g. parent responsiveness) without subsequent improvement in child speech production; (2) where speech production improvements are seen, these rarely extend beyond the target stimuli (lack of generalisation); and (3) participants are highly heterogeneous in their response to interventions.

Given there is not yet an established intervention tailored to improving motor speech in this population, we sought to design and create one, with the ultimate goal of examining the causal relationship between speech production skills and expressive language development. The intervention reported in this paper employed two techniques novel to language interventions for autistic children: video modelling and cued articulation.

Video modelling is a technique whereby a target behaviour is demonstrated via a pre-recorded video played to the learner via an electronic device, rather than through live demonstration. The person demonstrating the behaviour in the video (the model) can be a peer, an adult or the learners themselves (video self-modelling). Videos are designed to accentuate important features of the behaviour and remove distracting extraneous stimuli, and video modelling interventions may involve repetition of the stimuli to enhance learning. Several meta-analyses have concluded that video modelling can be effectively used to promote the acquisition of a variety of academic, social, communicative and functional skills in autistic children and adolescents [29–31]. To our knowledge, video modelling has not been investigated as a potential tool for speech production training; however, it has been used to promote spontaneous requesting via speech-generating devices [32] in participants with a similar profile to those in the current study.

Cued articulation [33] is one way of visually indicating how a speech sound is made, for those who do not find it easy to copy speech sounds. The rationale behind cued articulation is that each phoneme is accompanied by a hand gesture which provides a visual clue as to how and where the sound is made by the articulators, for example, a ‘p’ sound starts with rounded lips that open when the sound is released, and the ‘p’-cued articulation gesture is index finger and thumb creating a circle which then opens as the sound is made. Unlike manual imitation, speech sound imitation cannot be physically prompted, and because much of it occurs inside the mouth, it can also not be viewed. Cued articulation has rarely been tested in research studies but has been widely used by speech and language therapists (SLTs) in a variety of conditions including English as an additional language, hearing impairment, autism and speech sound disorders, despite this lack of empirical evidence [34].

The intervention was devised to encourage children to practise speech sounds with a parent, in order to increase their speech sound repertoire. It aimed to take into account specific features of autism and adapt typical approaches to speech skill training accordingly. High-quality intervention evidence for children with speech-motor difficulties is lacking [35]. Nevertheless, widely delivered interventions frequently include (a) the provision of high-quality multi-modal models of sounds to be imitated and (b) facilitating frequent practice of the sounds incorporating the principles of motor learning [36]. For a myriad of reasons, typical approaches may be problematic for autistic learners and need to be adapted.

Repeated modelling of sounds in the natural environment is designed to draw the child’s attention to how to articulate a given sound, often supported by additional visual cues such as the cued articulation signs. If a child is minimally verbal, parent-child interactions may not afford as many natural opportunities for the parent to model the sound. Briefly presented multisensory social input (e.g. sound and lip movement) may be less precisely perceived by autistic individuals [37]. By placing the speech sound model in a very structured repetitive video with no distractions in the background, we hoped to reduce the attentional load required to process the model.

Repeated practice is needed in order to master a specific motor skill. SLTs often achieve this by playing motivating interactive games with a child, e.g. a ‘fishing for sounds’ game where child and therapist take turns to lift up pretend fish with a magnet fishing rod, each fish having a sound symbol or picture. The person has to say the sound aloud when they have ‘fished’ it. Autistic children may find interactive games with an unfamiliar SLT aversive, or if learning difficulties are present, play-related tasks could increase the cognitive demands of the task (e.g. child struggling with fine motor aspects of ‘fishing for sounds’ game). Simplifying the task and removing the interactive aspect may thus benefit autistic children. Motivation is of course important, and it may be possible to replace the assumed social motivation with a child’s special interests, to motivate them to continue with speech practice. An example would be using video clips as a reward after attempting the target sound.

Importantly, the intervention was designed to be simple, portable and requiring no additional materials or reporting, given that engaging children in less preferred activities may be challenging enough for parents. It was thus designed to be delivered via a smartphone application (or ‘app’). Smartphones and tablets hold much promise as cost-effective, flexible and efficient delivery systems for a range of educational interventions, and reviews have demonstrated their effectiveness for autistic learners across a host of skills [38–42].

## Aims

The central aim of the current study was to develop and pilot an app-based speech sound intervention for minimally verbal autistic children, incorporating video modelling and cued articulation.

### Phase 1: Intervention development

The design phase involved two stages of formative evaluation, resulting in improvements to the app. The aims of this phase were as follows:

1. To seek feedback on the preliminary concept from a focus group comprising parents of autistic children with additional language difficulties and incorporate it into the initial app design
2. To briefly pilot a prototype of the app with a convenience sample and incorporate their feedback into the version used for preliminary pilot testing

### Phase 2: Preliminary pilot testing

The pilot study aimed to evaluate two important aspects of feasibility in a sample of minimally verbal autistic children: intervention acceptability and usability. Our pre-registered research questions were as follows:

1. Will parents rate this intervention as acceptable? Acceptability will be tested by simply counting the proportion of parent-child dyads who score greater than 24 on the parent satisfaction measure.
2. Will parent-child dyads comply with the intervention to a reasonable degree? Usability will be tested by counting the proportion of participants who spend a mean of > 12.5 min/week on the intervention.

We explored additional analyses to further understand usability:

1. Did parents comply with the intervention schedule (i.e. did they begin the intervention on time)?
2. Did parents comply with the test schedule?
3. How many intervention trials per week did the parents do (i.e. did they spend 5 min per day completing just one trial)?
4. Do any of the factors considered in this study explain whether parent-child dyads were ‘high’ or ‘low’ users of the app?

Finally, we aimed to collate and synthesise qualitative feedback regarding the app’s acceptability from parents.

## Method

This section first describes the intervention used in the pilot study, and how it was designed and modified with autism community input (stage 1 and stage 2 of consultation). In the second section, the pilot study methodology is described.

### Phase 1: Intervention development

#### Design process

Our iterative intervention design process comprised the following:

- Initial design
- Stage 1 consultation and app creation
- Stage 2 consultation and associated improvements to the app

The app was initially designed by the authors in collaboration with a team from the University College London (UCL)’s Computer Science Department. In March 2017, before coding for the app had begun, we carried out stage 1 of consultation (described below). Once a working version of the app had been created (May 2017), we trialled it on a small convenience sample of users (stage 2 of consultation, described below). Afterwards, an independent programmer was commissioned to carry out the recommended changes and solve highlighted technical problems, resulting in the prototype version of ‘BabbleBooster’, which was used for the pilot study. This version is described, followed by brief summaries of both consultation exercises and the improvements that resulted.

There is a growing awareness that high-quality autism research should directly involve autistic individuals as partners within a participatory framework. Fletcher-Watson et al. [43] advocated for ‘user-centred design with relevant stakeholders’ in their description of the design process for an app-based intervention game designed for young autistic children but discussed the challenges of facilitating full participation by the user group. In some cases, necessary input is sought from family members and experts in ‘participation by proxy’. Given our aim to create an intervention for minimally verbal autistic children, we engaged in participatory design with parents both during stage 1 of the consultation and for the pilot study, as they are the principal agents of delivering this intervention and best placed to advocate for their child’s communication needs.

#### BabbleBooster description

BabbleBooster was designed to deliver predictable and repetitive speech models via video modelling and with cued articulation. The app-play is parent-mediated, so parents are required to watch the stimuli with their children, encourage them to make the sound and then provide feedback on the sound in order to trigger the reward videos. Reward videos are designed with a gradient response, so a ‘good try’ at a sound (an incorrect attempt) will result in a lesser reward than an accurate response. The families were encouraged to make or upload their own reward videos, based on their understanding of the individual child’s specific motivators.

BabbleBooster was designed specifically for use in a case series design, whereby each participant acts as their own control and the outcome variable is tested repeatedly both before and during the intervention period. Each participant is given a personal intervention schedule comprising A (baseline) and B (intervention) weeks. BabbleBooster thus functions in one of two ‘modes’ depending on whether the participant is in an A or a B week:

- *Test mode*: this is during the baseline data collection period. The intervention itself is not accessible, but the test module is live. Once per week, the participants are prompted by text message to complete the test module.
- *Training mode*: this is during the intervention period. Both the intervention and the test module are live. The participants are expected to carry out the intervention as per instructions, plus complete the weekly test module as above.

Each participant is likely to have a unique profile of speech skills, meaning that targets need to be individualised. Nine probe phonemes were allocated to each child at the start of the intervention by following the ‘Sound Target Protocol’ (see Additional File 4), of which three were allocated as intervention targets and six were controls. Each week, the test module comprised nine single trials of all nine probe speech sounds. The untrained sounds were used as a control to compare with trained sounds (to investigate whether there was a systematic relationship between any improvement in speech production and the intervention) and to assess whether any improvements generalised to other sounds. Weekly test score was calculated as a percentage, representing the number of phonemes correctly produced out of nine.

For each of the three *target speech sounds*, there is a set of learning stimuli which comprise the following:

- *Mandatory content*: this is an unchangeable content, such as the auditory model of the sound and the cued articulation video.
- *Customisable content*: which can be added to, removed and changed as much as desired by the child (with help from the parent). For example, the app comes loaded with images of items beginning with ‘t’ for the ‘t’ target (e.g. tiger), but the child may have a favourite toy called ‘Timmy’ or a family friend called ‘Tania’—images of these specific items can be transferred onto the app to create a more meaningful personalised set of stimuli. Example screenshots are provided in Additional File 1.

In training mode, after watching the learning stimuli, the child is prompted to attempt the speech sound. Children can use the video capture part of the app as a mirror whilst speech attempts are being recorded and have the opportunity to play back and review their speech attempts. Parents then press one of three buttons to assign a rating to the attempt, in accordance with Table 1. Depending on the parent feedback, the child is either presented with a customisable reinforcement video as a reward, or another attempt begins. The app records progress made by the child and determines whether mastery criteria have been fulfilled and whether a new target can be selected or the existing target should continue.

**Table 1:** BabbleBooster parent rating buttons

Button	Meaning	Example	Consequence
Yes	Child has produced elicited sound accurately	Child is asked to say /b/ and they say /b/	‘Well done’ video
Good try	Child tried to make a sound but did not make the target sound	Child is asked to say /b/ and they say /w/	‘Good try’ video
Try again	Child does not attempt to make any sound	Child is silent/shouts/cries	No video clip

**Fig. 1:**
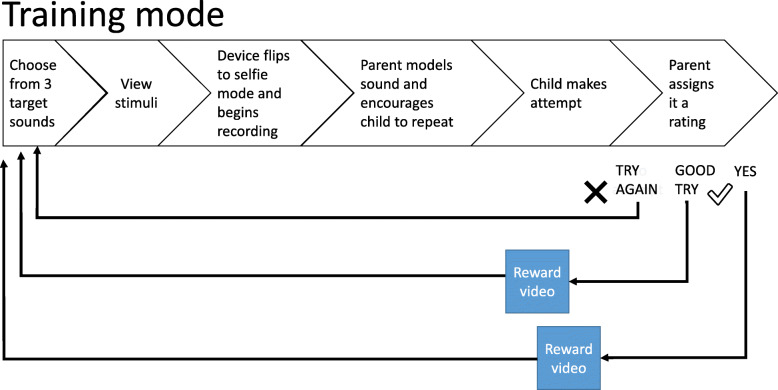
Depiction of one intervention trial

Figure 1 depicts how a single ‘trial’ of the intervention works.

#### Consultation stage 1

An initial version of the app was presented at a focus group in March 2017 with four parents whose autistic children have co-morbid language difficulties (referred to as participants L, E, R and A). A fuller description of the focus group is provided in Additional File 2. Their input contributed to the app prototype and is briefly summarised below.

##### Technology

All parents reported that mobile and tablet devices were inherently motivating for their children, with the most commonly used function being to access video content online (e.g. via YouTube). The content was often esoteric, user-uploaded and specific to the child’s special interests (e.g. people going on waterslides, opening toys).

##### Aim

All liked the idea of the app and the mirror function. Parents suggested that having images which match the sounds would make the learning more functional. Parents would like to have input on the initial sound selection process.

##### Time commitment

All parents agreed 5 min per day is an achievable target.

##### Cued articulation aspect

Only participant A had heard of this approach, but when her daughter was minimally verbal, she had found it very helpful in progressing speech skills.

##### Video modelling aspect

All agreed this would be good. Participant R said it was hard to get her son to look at her whilst she modelled language. She believes this is why he found PECs (a picture exchange communication method) easier than Makaton (a simplified form of sign language, requiring learners to copy manual signs from adult models).

##### Parent feedback on child productions

Parents unanimously disliked the proposed red ‘no’ button, reporting that their children were very sensitive to ‘getting things wrong’. They suggested changing it to a ‘try again’ button and altering the colours.

##### Reinforcing videos

All parents agreed that customisable content is a must-have feature of the app. Various ways of supporting parents to create content were discussed, for example, providing a parent idea sheet or ‘how to’ videos.

In summary, changes to the app from this stage of consultation were as follows:

- Rather than just providing the sound and a letter symbol in the sound modelling phase, this was changed to three images corresponding to the sound (e.g. for ‘b’: ‘baby’, ‘ball’, ‘biscuit’). These can be replaced or exchanged by parent customisation.
- Rather than presenting parents with three choices for feedback buttons (‘yes’, ‘good try’ or ‘no’), this was changed to ‘yes’, ‘good try’ or ‘try again’, and red and green colours were removed.

#### Consultation stage 2

A second feedback phase occurred prior to launching the pilot study. In May 2017, a convenience sample was invited to try the app, over the course of a week. The group included parents of children with additional needs, who were or had been preverbal. Due to time and budget constraints, this convenience sample only contained one parent of an autistic child. Given that the aim of this stage was to identify technical issues rather than shape the design, this was deemed acceptable. A fuller description of the test phase is provided in Additional File 3. This process highlighted the technical glitches and generated further improvements to the layout.

Summary of changes from this stage is as follows:

- Addition of a replay button so the attempt videos can be re-watched
- Addition of in-app camera to take photos of stimuli directly from the customisation menu to aid customisation

### Phase 2: Preliminary pilot testing

#### Participants

Participants were 19 minimally verbal autistic children (three girls, 16 boys) who met the following criteria:

- Parent reported fewer than 10 sounds
- Parent reported fewer than 20 words
- During observation at visit 1, fewer than five words spoken

The children were aged 47 to 74 months at visit 1 (mean = 60, SD = 7) with a confirmed diagnosis of autism. The following exclusions were applied at the initial screening: epilepsy; known neurological, genetic, visual or hearing problems; and English as an additional language.

**Fig. 2:**
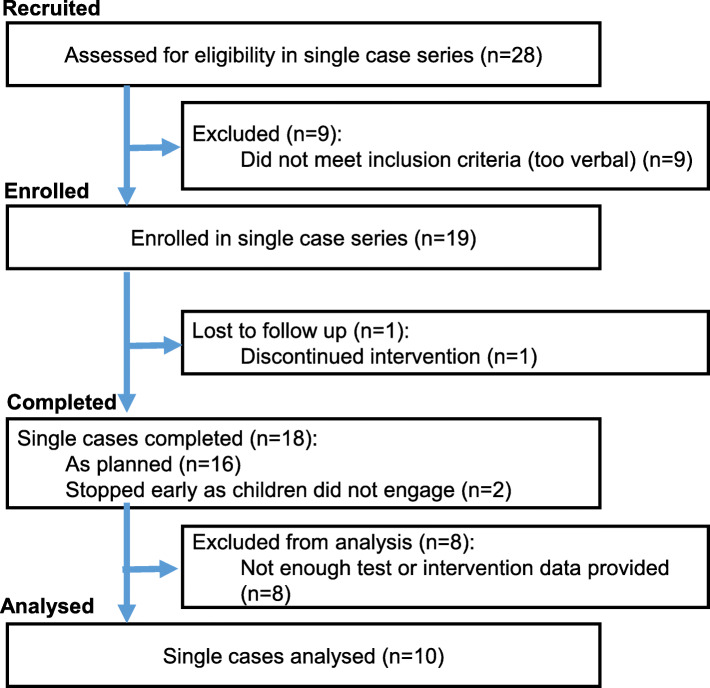
Recruitment flow chart

Children were initially recruited via social media, local charities, independent therapists and a university-run autism participant recruitment agency, and all took part in a larger longitudinal study [12]. The goal of the larger study was to investigate predictors of expressive language development in autistic 3–5-year-olds who were minimally verbal at study inception. Children were visited three times in their homes prior to the current study (see Fig. 4). As per Fig. 2, children who remained minimally verbal by the fourth assessment wave (visit 1 of the current study) were invited to participate in the intervention.

Parents reported 17 participants to be White, one to be Asian and one to be mixed race. The formal education levels of the primary caregivers were distributed as follows: eight completed high school, eight completed university education and three completed post-graduate studies or equivalent. Eighty-eight per cent of parents reported that their child had an Education Health and Care Plan, a legal document that specifies special educational support required for the child, at visit 1.

Figure 2 describes the process through which participants were selected for the study.

#### Procedure

Children were visited in their homes by the first author in two sessions (visit 1 and visit 2), which were separated by 4 months each (mean = 4.0, SD = 0.3). A token of appreciation (small toy or £5 voucher) was provided following each visit.

At *visit 1*, each participant received a new Samsung Galaxy Tab A6 tablet containing the app, unless parents expressed a preference to use the app on their own Android device (*n* = 3). One participant received a comparable second-hand Nexus 7 tablet. Parents were given a demonstration of the app by the experimenter and an information pack explaining how to download and use the app. The probe phonemes were selected by following the ‘Sound Target Protocol’ (see Additional File 4), and each parent-child dyad was informed of their randomly allocated intervention start date. Probe phonemes are the nine sounds that are elicited each week in the baseline and intervention period. They also form the list from which initial three target phonemes are drawn for the intervention. Probe phonemes remained the same for each participant throughout the study, whereas the target phonemes for the intervention could vary over time according to specific mastery criteria. Probe phonemes were not manipulated as part of the experiment; rather, they were a necessary feature to accommodate the fact that each participant has a unique profile of speech-related difficulties.

At *visit 2*, parents completed a post-intervention questionnaire (see Additional File 5) in order to objectively analyse the user experience of this intervention. It contains a grid of 10 questions regarding the usefulness and user-friendliness of the app, which can each score between one and four points, generating a score ranging between 10 and 40, with 40 representing the most positive rating of the app possible. Additionally, the questionnaire contained four open-ended questions regarding the strengths and weaknesses of the app.

At *both visits*, a battery of language-related measures was taken, some of which were designed as secondary outcome variables (parent-reported measure of expressive language and an observed measure of the range of speech sounds made during a language sample, ‘consonant inventory’) and others related to a broader longitudinal study (of which these visits were time points 4 and 5). As part of this battery, at visit 1, questionnaires on AAC use and educational placement were completed. All participants were free to take part in as much or as little additional therapy as they chose during the study, and this information was recorded via parent questionnaires at both visits.

**Fig. 3:**
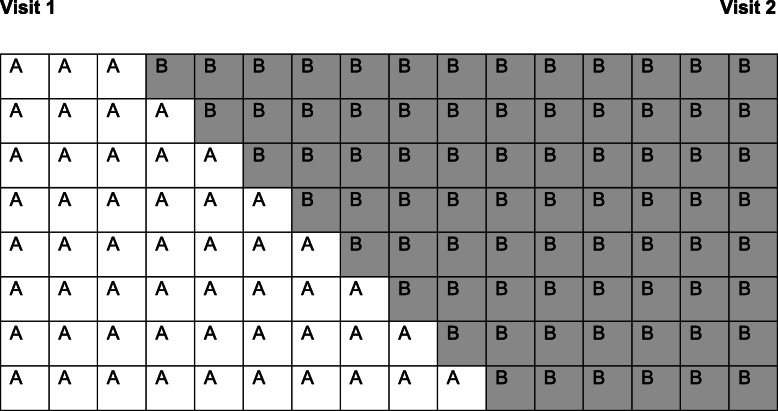
All possible permutations of baseline (A) And Intervention (B) weeks

*Between visits 1 and 2*, text message reminders were sent to parents to remind them of the weekly obligation to complete the test module (a ‘probe day’), and if necessary, missed probes were rearranged for the following day. There were 16 weeks between visits 1 and 2, and parents were randomly allocated to one of eight possible intervention schedules, as illustrated in Fig. 3. On the intervention start date, parents received a reminder text. Thereafter, parents were asked to play with the app for 5–10 min per day for 5 days a week. This resulted in children carrying out the intervention for between 6 and 13 weeks.

Throughout the baseline and intervention period, data from the app were uploaded regularly to a secure server accessed by the experimenter. These data comprised the following:

- Information on time, type and duration of app usage (e.g. participant 1 used the app for 35 s on test mode at 13:05 on 21 December 2018)
- Videos and parent ratings of probe trials each week
- Videos and parent ratings of the intervention trials during the intervention phase

**Fig. 4:**
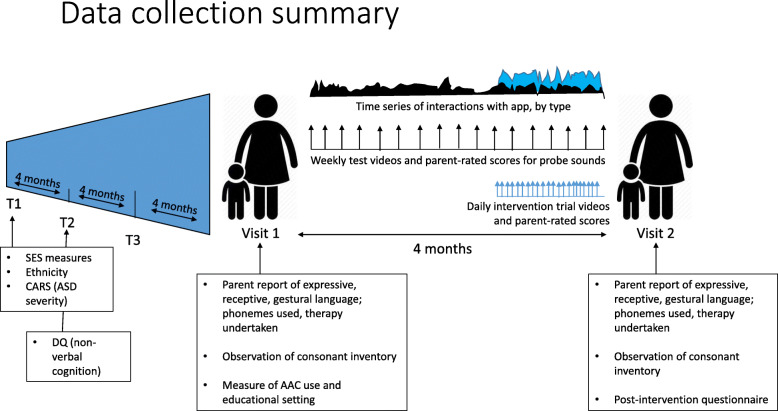
Summary of the data collection (longitudinal study and current study). AAC, augmentative and alternative communication; ASD, autism spectrum disorder; CARS, Childhood Autism Rating Scale; DQ, developmental quotient (developmental age/chronological age); SES, socio-economic status

Additionally, as the participants are drawn from a previous longitudinal study (see [12] for further details), further background measures, which were gathered between 8 and 12 months prior to the current study, were also available to characterise the sample. A summary of all the relevant data collected (including the larger study) is provided in Fig. 4.

#### Measures

The background measures gathered for each participant are summarised in Table 2.

**Table 2:** Background measures

Measure	Time	Description
Parent SES	Time 1	A parent questionnaire determined the highest education qualification (averaged across parents if applicable), as a proxy for socio-economic status (1 = high school, 3 = post-graduate studies).
Autism symptom severity	Time 1	The experimenter completed the Childhood Autism Rating Scale (CARS) [44]; higher scores indicate greater autism symptoms.
Non-verbal cognition	Time 2	Visual reception and fine motor subtests of Mullen Scales of Early Learning [45] were transformed into a developmental quotient (developmental age/chronological age)
Receptive language	Visit 1	Number of words understood according to the British Communicative Development Inventory [46], completed by parents.
Expressive language	Visit 1	Number of words spoken according to the British Communicative Development Inventory [46], completed by parents.
AAC use	Visit 1	A parent questionnaire determined whether the child regularly requests 10 or more items using any augmentative alternative communication system (coded as yes/no).
Total therapy	Visit 2	A parent questionnaire was used to describe and quantify all therapies undertaken by the child in the previous 4 months (h/week).
Speech and language therapy	Visit 2	A parent questionnaire was used to describe and quantify all speech and language therapy undertaken by the child in the previous 4 months (h/week).

Time 1 = 12 months prior to visit 1. Time 2 = 8 months prior to visit 1

The accessibility analysis in this paper will focus on (a) the post-intervention questionnaire and (b) the information on time, type and duration of app usage. Analysis of efficacy measures such as weekly probes and pre- and post-intervention variables is provided in [47]. The random allocation of intervention schedules and repeated probing of an outcome measure are both key components of the study design, which is explained in greater detail in the efficacy analysis paper [47].

#### Data analysis

##### Preregistered questions

The analysis plan was pre-registered on Open Science Forum at https://osf.io/9gvbs; the acceptability and usability hypotheses were as follows:

1. Acceptability: More than half of the participants given the intervention will rate the app favourably via the feedback form, as defined by a score of more than 24/40 on a 10-question feedback form, where each question can be answered from one (not favourable) to four (highly favourable). This hypothesis will be tested by simply counting the proportion of parent-child dyads who score greater than 24 on the parent satisfaction measure.
2. Usability: More than half of the participants given the intervention will comply with the intervention to a reasonable degree, as defined by an average of more than 12.5 min per week engaging with the app. This threshold was based on the instruction for parents to spend 5 min per day for 5 days a week using the app with their child. This totals an average of 25 min per week, which would define 100% compliance. Based on previous studies [48], we rated 12.5 min per week (50% compliance) to represent the lower threshold of ‘reasonable compliance’. This hypothesis will be tested by counting the number of participants who spend a mean of > 12.5 min/week on the intervention and dividing by the total number of participants.

Multiple other aspects of compliance were investigated using the available data to answer the following questions:

1. Did parents comply with the intervention schedule (i.e. did they begin the intervention on time)? This was evaluated by calculating the number of weeks of delay (vs. scheduled intervention start) for each participant.
2. Did parents comply with the test schedule? This is important for the chosen intervention efficacy evaluation method and was evaluated by counting the proportion of planned weekly test trials that took place for each participant.
3. How many intervention trials per week did the parents do (i.e. did they spend 5 min per day completing just one trial)? This was a mean weekly trial count for each participant.
4. Given that the participants separated into ‘high’ users, who provided enough test data for analysis purposes, and ‘low’ users, who completed fewer than 4 weeks of test probes, we asked if any background variables or other factors could explain these groupings. This was evaluated via a series of *t* tests comparing the values for each group.

Finally, feedback regarding the app from parents was aggregated from various sources (texts, emails, written answers to open-ended questions on the App User Questionnaire, notes made by the first author from verbal comments made by parents at visit 2 following the intervention). The first author analysed this data using thematic analysis [49], in order to glean further qualitative information regarding acceptability and potential avenues for improvement.

## Results

### Acceptability: parent satisfaction

The acceptability questionnaire was completed by 89% of participants.

Table 3 outlines the key characteristics of each of the 19 participants and their acceptability score. Over half of the participants were ‘high’ users (defined as providing greater than 66% of test trial data). Two participants dropped out during the trial, one was lost to follow-up and the remaining six participants engaged with the app but not enough to produce analysable data (fewer than 4 weeks of test data and fewer than five intervention trials). Of these six participants, three did not find the technology motivating, one had ongoing health problems; one had a technical issue with the app and one made language progress via another therapy during the course of the intervention so did not engage with the app.

**Table 3:** Descriptive statistics for the whole sample

ID	Sex	Visit 1 age (months)	Visit 1 RCDI (words)	Visit 1 ECDI (words)	User type	Device used	Acceptability score	Visit 1 therapy hours per week, total (SLT)
1	M	58.9	314	9	High	Provided	35	9 (1)
2	M	56.4	38	0	High	Provided	30.5	6 (0)
3	F	55.8	5	0	High	Provided	29	39 (0)
4	M	59.5	282	1	High	Provided	27	2 (1)
5	M	50.3	37	1	Minimal	Provided	29	0 (0)
6	M	62.5	103	6	d/o	Provided	NA	0 (0)
7	M	60.4	290	0	High	Provided	34	0 (0)
8	M	46.9	171	1	Minimal	Provided	22	1 (1)
9	M	54	NA	NA	l/f	Own	NA	NA (NA)
10	M	58.7	55	0	High	Provided	29	7 (1)
11	M	73.5	68	0	High	Provided	35.5	2 (2)
12	M	53.6	212	19	High	Provided	33	1 (1)
13	M	63.6	116	13	Minimal	Provided	25	0 (0)
14	M	72.8	404	8	Light	Provided	29	0 (0)
15	M	67.7	406	18	Minimal	Provided	29	1 (0)
16	F	68.3	245	0	d/o	Own	26	0 (0)
17	M	55.1	189	0	Light	Provided	29	0 (0)
18	F	68.6	337	0	High	Provided	35	0 (0)
19	M	61.8	8	5	High	Own	25	0 (0)
**Mean**		**60.4**	**182.2**	**4.5**			**29.5**	**3.7 (0.4)**
**SD**		**7.3**	**137.9**	**6.3**			**10.0**	**8.9 (0.6)**
**Minimum**		**46.9**	**5**	**0**			**22**	**0 (0)**
**Maximum**		**73.5**	**406**	**19**			**35.5**	**39 (2)**

Acceptability score is out of 40

*d/o* drop out, *l/f* lost to follow-up, *ECDI* Expressive Communicative Development Inventory, *RCDI* Receptive Communicative Development Inventory

The pre-registered hypothesis that over half of participants would assign the app an acceptability score of over 24 was confirmed. In fact, participants gave the app a mean score of 29.5 out of 40, and only one participant rated it below 24 (see Additional File 6 for score breakdown).

To supplement this result, we briefly summarise the qualitative feedback gathered via feedback forms and verbal comments made by parents at visit 2.

The overall premise and main features of the app were well received. One parent wrote, ‘We are working on single sound production in ad hoc way and it is good to have a framework/system to focus us.’ Another wrote, ‘Previously have focused on whole words, this strips it back to a more basic skill, which I think is what we need.’

Parents reported the app was quick to do, simple and accessible, facilitating practice little and often. Stimuli were clear and predictable, which parents felt was a strength. Parent quotes include that it was a ‘short focused activity that we could fit into everyday life, simple and easy to use’ and ‘my son enjoyed the predictability’. Many parents reported that their children particularly liked the video modelling stimuli.

Most parents reported that their children were specifically engaged by the mirror function (being able to watch themselves on the screen during the speech trials and having the option to re-watch these videos, e.g. ‘the selfie aspect was something I had not tried previously and my child responded well to it’. One parent suggested that in a future version, the videos could be side by side with the selfie screen during practice attempts. However, for a few parents, this feature was felt to have a negative impact in their child’s engagement with the app (one parent reported a more general issue that their daughter had with mirrors and asked if the mirror function could be made optional). Another parent suggested masking the eyes as they felt their child did not like looking at their own eyes but would have found viewing the mouth useful.

Feedback also highlighted five main areas that could improve acceptability:

1. *Better training and support with customisation*. Although customisation was a popular feature and reported to be easy to do, several parents said that they found it difficult to source the customised stimuli. This problem was compounded by the fact that most users were not using BabbleBooster on their ‘own’ phone and thus did not readily have access to their own photo library and usual apps. Several parents suggested in a future trial that we make a library of popular videos and images. One parent reported frustration that the app could not interface directly with YouTube, since that was where all her child’s videos were and it had the functionality that her child was used to (e.g. volume controls, fast forward buttons).
2. *Modifications to the test mode to make it more engaging*. The test mode did not have any built-in reward videos, and many parents said this made it difficult to engage their children, particularly as they were first exposed to only the test mode, during the baseline phase. Furthermore, several parents reported that the probe contained too many targets (nine), and three would have been more manageable.
3. *Solving technical problems and limitations*. Technical problems were an impediment to participation in that for a couple of users, the app would take a long time to initiate or would fail to login. This made it hard to plan therapy time and left the child and parent feeling frustrated. A few users reported that crashes led to the need to re-customise the stimuli, which was time-consuming. There were reports of recordings stopping mid-video, leading to a lack of reward and frustration. From a research perspective, we estimate that some data has been lost through technical faults, preventing analysis of the full dataset. For 15% of the parent ratings submitted in the test phase, the accompanying video is missing due to technical problems. It is not possible to estimate how much more data may have been lost due to incomplete trials. In this trial, it was not financially feasible to provide the app in OS (suitable for iPhones/iPads) as well as Android format, and this had numerous disadvantages. Two children were motivated by technology but had aversion to the new device because it was not their usual one and did not have all the apps they had on the other one. The new device became linked with work/demands and thus not motivating.
4. *Introduction of more variety and visual interest in intervention presentation*. One aspect of this feedback is similar to point 3 above: parents requested that the app look and sound more game-like, e.g. tones, jingles, cartoons, glitter effects, colours and animations. A specific suggestion was the incorporation of letters in the app itself (some members of this cohort had a special interest in letters). Some parents reported an initial high level of engagement and then a fatigue effect, which was revived once targets were substituted.
5. *Introduction of progress feedback for parents*. Many parents stated they would like quantitative progress feedback to encourage them to continue with the activities, e.g. percentage of attempts that were correct, minutes spent using the app and progress towards goals.

In sum, a more ‘game-like’ app that can run on participants’ own devices and is easier to customise with a wider range of personally relevant stimuli is a key factor that would enhance user experience and could be implemented in future trials.

### Usability

In order to consider whether the second pre-registered hypothesis can be confirmed, we have presented usage data in Table 4.

**Table 4:** Usability data for the ‘high’ user group

ID	Planned weeks of intervention	Delay to intervention start (weeks)	Actual weeks of intervention	% Weeks adhering to intervention (%)	% Total test trials completed (%)	Intervention trials/week	Min/week during intervention	Min/week during baseline
1	12	3	6	50	69	18	9.04	8.64
2^a^	10	3	5	50	71	0.6	8.72	6.25
3	7	0	4	57	71	9.8	11.61	6.38
4^a^	8	0	7	88	94	3.7	3.88	2.04
12	11	1	7	64	76	6.4	5.32	2.21
10	12	0	9	75	88	15.9	35.85	14.47
11	9	1	7	78	75	14.1	13.65	2.92
19	7	3	3	43	88	76	20.74	8.06
18	6	2	2	33	100	1	1.44	4.02
7	13	1	10	77	83	6.3	11.63	11.11
**Mean**	**9.5**	**1.4**	**6.0**	**61**	**82**	**15.2**	**12.2**	**6.6**
**SD**	**2.5**	**1.3**	**2.5**	**18**	**11**	**22.2**	**9.9**	**4.1**
**Min**	**6.0**	**0.0**	**2.0**	**33**	**69**	**0.6**	**1.4**	**2.0**
**Max**	**13.0**	**3.0**	**10.0**	**88**	**100**	**76.0**	**35.8**	**14.5**

Actual weeks = planned weeks minus delay minus missed weeks (where intervention not used at all for 1 week)

^a^Participants who completed very few trials in the intervention but were included in this group due to their adherence to the test schedule

From this table, it is apparent that only three participants used the app for longer than 12.5 min per week of intervention (fewer than half of the expected time on task); therefore, the hypothesis is not confirmed. Time spent on intervention and the number of trials per week of intervention were both highly variable.

Other usability metrics are also presented; notably, that of the ‘high’ users, 82% of all test trials were completed, indicating that compliance with the test element of the trial was good in this subgroup. It was also not time-consuming, taking a mean of 6.6 min to complete per week. Adherence to the intervention schedule was also reasonable at 61%, with a mean delay to starting of 1.4 weeks.

### Characteristics of the ‘high’ user group

Given that approximately half of the participants engaged successfully with the app to some degree (*n* = 10), and nine participants did not, we present an exploratory side by side analysis of group characteristics in Table 5, to explore whether background characteristics like family socio-economic status or child symptom severity influenced the use of the app. This analysis also highlighted the limited amount of special clinical services these families were receiving, on average fewer than 2 h per week. The total therapy hours/week variable is skewed by one high value (57 h vs. mean of 1.3 h/week for all other participants).

**Table 5:** Descriptive statistics describing the demographic features of the high vs. low user groups

	‘High’ group					‘Low’ group				
	*n*	Mean	SD	Min	Max	*n*	Mean	SD	Min	Max
Age at visit 1 (months)	10	60.7	6.0	53.6	73.5	9	60.1	8.9	46.9	72.8
Parent SES	10	1.8	0.7	1	3.5	9	1.6	1.0	1	3.5
Autism symptom severity (− 12 months)	10	43.0	5.1	35	49	9	42.3	4.9	37.5	52.5
Non-verbal cognition DQ (− 8 months)	10	0.4	0.1	0.13	0.56	9	0.3	0.1	0.18	0.52
Receptive language at visit 1 (words)	10	161	137.9	5	337	8	209	136.0	37	406
Expressive language at visit 1 (words)	10	3	6.3	0	19	8	6	6.7	0	18
% AAC user	8	60%				8	60%			
Total therapy (h/week) (visit 2)	10	7.0	17.5	0.0	56.6	8	1.1	2.1	0.0	6.0
SLT (h/week) (visit 2)	10	0.6	0.7	0.0	2.0	8	0.2	0.3	0.0	1.0

*AAC* augmentative and alternative communication, *DQ* developmental quotient (developmental age/chronological age), *SES* socio-economic status, *SLT* speech-language therapy

## Discussion

This is an acceptable intervention as judged by the pre-registered analysis of post-intervention questionnaires. Qualitative results reveal strengths in the study and app design and areas for further development that largely focus on solving technical issues and ease of customisation and gamification. This finding indicates that the app intervention has numerous features which parents and children liked and fostered engagement, and it has potential for future development.

Usage figures, however, presented a more mixed picture. Participants polarised into those who engaged with the app to a minimal degree (*n* = 9) and those who adhered well to the test schedule (*n* = 10). Of these 10 children, intervention usage figures were highly variable (in minutes spent and trials per week), but reasonable overall adherence to the intervention schedule was observed. Due to the unique features of this intervention, it is difficult to compare these usage figures to others in the literature. Adherence to parent-mediated autism interventions tends to be evaluated on criteria such as training session attendance [50] or how many learnt strategies are employed by parents at subsequent observations (e.g. [51]). App-based autism interventions are usually designed to be independently accessed by users (e.g. [52]). The shared app-play here resembles more closely the off-line ‘homework’ allocated by speech-language therapists for children with speech sound disorders; however, the developmental and behavioural profile of children in this cohort is more challenging. A useful comparator is [53] which describes a feasibility trial of an app-based parent communication training for children with motor and communication disorders. Parents were required to upload and annotate videos of themselves interacting with their child at regular intervals and received remote coaching on their use of key strategies. The attrition rate was 44%, and participation levels fell short of the target (target = 39 sessions, median = 26, range = 5–33). Parents reported that they found the intervention useful but cited time pressures and technical problems amongst reasons for lower engagement.

Many who found the app acceptable still dropped out or engaged only minimally, and it is important to consider the reasons why this happened.

This was a pilot study run on a minimal budget, and consequently, numerous practical challenges were encountered, particularly in resolving technical difficulties. Loss of data and frustration leading to avoidance also explains why some children and families were minimal users. We believe that these problems would be surmountable if a professional app company were to be engaged. This pilot has highlighted the importance of having enough memory capacity on the chosen device, which must be weighed up against cost considerations for any future trial. A lack of cross-platform approach led to limitations in device choice; without a doubt, using participants’ own phones would have been more effective. In a future trial, we would strongly recommend a cross-platform approach, so that participants can use their own devices.

Thematic analysis of qualitative feedback received from parents highlighted several key areas for improvement. Parents reported that additional support with customisation would be beneficial. Some of the difficulties stemmed from parents not using their usual devices for the intervention (due to the need to use a specific android device). This meant that they did not have direct access to their personal photo libraries. We had posited that with cloud-based file repositories and online access to limitless content (e.g. YouTube or Google Images), this would not be problematic; however, it appears to have influenced the degree of customisation which took place. The only official gauge of how much customisation occurred is by measuring minutes spent on the customising screen; however, this gives a limited impression of how much the stimuli were changed. In a future trial, we would recommend the data capture to give more detailed information of this or that parents record their customisation activities in a diary format. In addition, a library feature and better interfaces with popular video sharing apps would enhance the usability for parents, although a preliminary feedback exercise may be necessary to ascertain which content would appeal to users.

A second area for improvement was the need for gamification, particularly with the test module, which parents found less engaging than the intervention. Overall, the test phase was a stumbling block for many users and could have been responsible for several of the participants dropping out or not adhering to the intervention. It comprised nine probes, which parents suggested was too many. This number was chosen to provide enough items to demonstrate any improvement within participant over time, incorporating trained and untrained items. It was designed to be a ‘cold probe’ in order to provide evidence of an improvement in the target skill if there was one; therefore, no feedback was provided on speech attempts. An improvement would be to incorporate non-contingent rewards into the test phase, in order to ensure the children’s first exposure to the app was associated with the fun aspects that appear later in the intervention phase (i.e. reward videos). As BabbleBooster was a low-budget prototype, there was little scope to ‘gamify’ the app by incorporating visual effects such as spinning images and sound effects, but these may have also helped engage children from the outset.

Gamification of the intervention trials was also called for by parents. This would be a key area for refinement if this app is developed further. Amongst potential solutions are to intersperse targets with mastered items (although this may have to be a non-speech task depending on the children’s ability level) or to have more targets but rotate them frequently, perhaps at the syllable level so instead of just working on ‘b’, work on ‘bee’, ‘boo’ and ‘bah’. The need to individualise and differentiate activities is common in app-assisted autism interventions [54].

The final recommendation from parents was to incorporate a feedback mechanism, in order to inform and motivate families during the intervention. This was an initially planned feature that had to be disabled in the final version of the app due to cost constraints (a reworking of the app by the independent developer to resolve technical issues identified at stage 2 had caused the feedback mechanism to stop working, and no further funds were available to reinstate it). In future trials, this should be reinstated.

Parents of autistic children experience higher levels of stress [55–57], and thus, fitting an additional therapy task into daily life could also be challenging. The occurrence of family illness, carer chronic health conditions, siblings with additional needs and difficult transition periods between educational settings and school holidays were amongst the many barriers to adherence faced by this cohort. Parent-mediated speech and language therapy is often suggested, and digital tools such as BabbleBooster are designed to make this more feasible; however, we must be realistic that even this will be too much for some families, given their circumstances. Relatively few studies have analysed parent intervention adherence in families with a minimally verbal autistic child (e.g. [58]), and this is crucial to understanding what intervention approaches are likely to enhance parent co-operation.

Finally, we should recognise that app-based therapy is also not for everybody and that is especially true in this very heterogeneous group. Three of the children’s families reported that they were just not interested in technical devices. In these cases, future studies could consider whether the principles of the intervention design could be applied through different media. An improvement for future studies could be to evaluate technology use, familiarity and preferences amongst participants (parents and children) prior to an app trial. In the case of this study, children were recruited from a larger longitudinal study using pre-registered inclusion criteria, which did not include information regarding technology preferences, although the information materials available to parents as part of the consent process did explain that the intervention would be app-based.

This study was not adequately powered to examine associations between background measures and ‘high’/‘low’ group membership. Future studies could test these relationships or seek to identify other factors which could be important predictors of intervention compliance in this population, such as parent physical and mental health, employment, confidence in using technology or delivering therapy.

Considering the challenges identified above, a future trial could improve the technological and motivational aspects of the app. It is also possible that asking other significant adults (such as grandparents, learning support assistants) to use the app with the child in different settings could be an acceptable solution, in families where adherence to intervention is not feasible. More detailed predictions could be made regarding usability metrics, and data should be collected on customisation activities, in order to gauge whether the degree of engagement with customisation was a factor in subsequent acceptability and usage scores.

### Limitations

Like many feasibility studies, we evaluated participant satisfaction using a bespoke questionnaire, tailored to the key components of the intervention (e.g. [59, 60]). There are thus no appropriate benchmarks or norms available for our acceptability measure. Future studies could combine our highly informative bespoke measure with a commonly used generic intervention evaluation measure such as the Behaviour Intervention Rating Scale (BIRS; [61]). The BIRS is a 24-item inventory using a 6-point Likert-type scale (‘strongly disagree’ to ‘strongly agree’) and addresses acceptability and perceived efficacy. Equally, the pre-determined threshold to assess acceptability on this measure of 24/40 was set arbitrarily, whereas if a generic evaluation measure had been used, the threshold could be more robustly justified and compared with other studies.

Secondly, no fidelity measures were taken during the parent training aspect of this study (e.g. checklists of training topics or video coded analysis of parent training session). This was deemed unnecessary given the simplicity of the app and provision of a detailed manual, but it may be useful in a future study. Finally, thematic analysis of qualitative feedback from parents was evaluated subjectively by the first author alone. In future studies, an experimenter from outside the study could gather qualitative feedback using semi-structured interviews in order to reduce bias, and themes derived from transcribed interviews could be reviewed by another experimenter to ensure convergence.

The study aimed to incorporate user-centred design into the creation of the app, via a focus group (consultation stage 1). This phase did not lead to significant changes to the app, yet a wealth of proposed changes resulted from the pilot. This may suggest an ineffective consultation process, perhaps it was not done in sufficient depth or at the right time in the design process. These aspects were constrained by the timeline and budget for the study. Some aspects in need of improvement (such as technical problems which only became apparent after several weeks of video downloading) could not have come to light until the app was in daily use.

## Conclusion

This study reports a first attempt to develop and pilot a customisable app to develop speech production skills in minimally verbal autistic children, using video modelling and cued articulation to demonstrate where and how speech sounds were made, and video capture to record the child’s production efforts. Overall, parents reported that a structured focus on improving speech skills was welcome and reported that the app and the intervention design were acceptable. Nevertheless, parent compliance with the intervention schedule was highly variable and parents delivered about half of the recommended trials. Whilst technical issues with software and device may explain some of this, the demands of family life may make parent-mediated interventions more challenging for this population. A better understanding of how best to facilitate engagement in therapies is a priority for future research. Future research should aim to leverage the valuable lessons learned in the current study, in order to further develop and test app-based interventions for this hard-to-reach and underserved population. In particular, future work should investigate the impacts of duration, frequency and intensity for app-based speech interventions.

## Supplementary Information

**Additional file 1.** Learning stimuli (left: customisable; right: mandatory).

**Additional file 2.** Stage 1 Consultation.

**Additional file 3.** Stage 2 Consultation.

**Additional file 4.** Sound Target Protocol.

**Additional file 5.** Acceptability Questionnaire.

**Additional file 6.** Score Breakdown of Acceptability Questionnaire.

## Data Availability

The datasets used and analysed during the current study are available from the corresponding author on reasonable request.
