# Supplementary figures and images for: Feasibility of an app-based parent-mediated speech production intervention for minimally verbal autistic children: development and pilot testing of a new intervention
Source: Pilot Feasibility Stud. 2020 Nov 25;6:185. doi: 10.1186/s40814-020-00726-7 (PMC7687695; doi:10.1186/s40814-020-00726-7)

**Additional File 1**


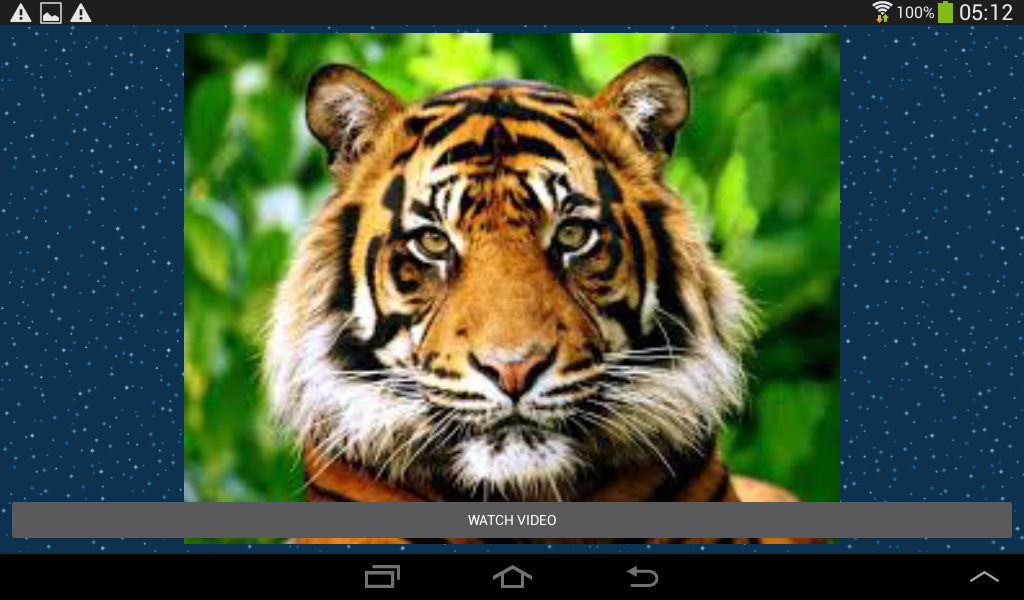

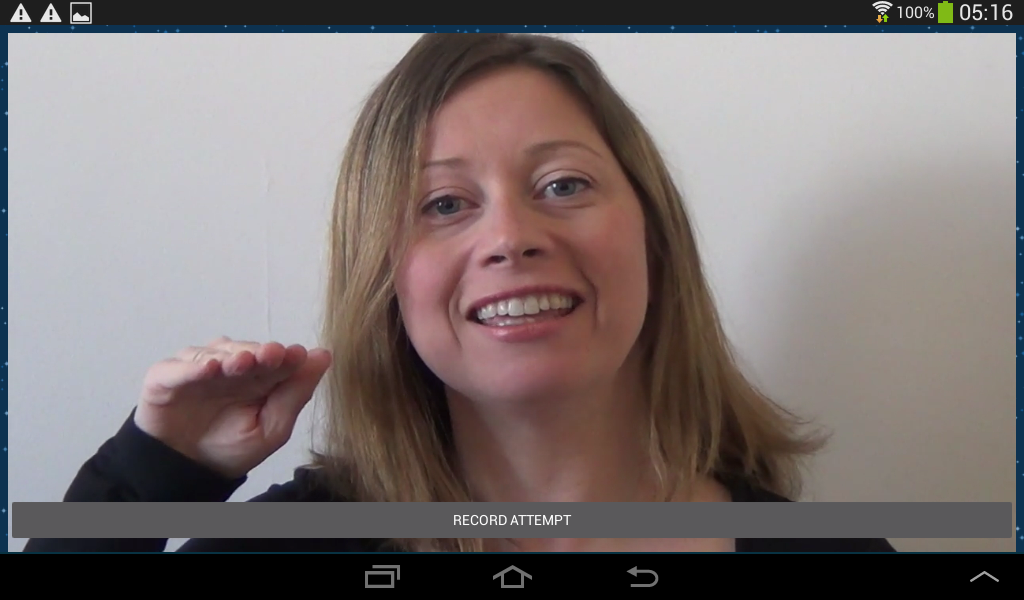


*Learning stimuli (left: customisable; right: mandatory)*

Supplement: Supplementary file 1 — Additional file 1. Learning stimuli (left: customisable; right: mandatory). [file 40814_2020_726_MOESM1_ESM.docx]
