# Supplementary material for: Feasibility of an app-based parent-mediated speech production intervention for minimally verbal autistic children: development and pilot testing of a new intervention
Source: Pilot Feasibility Stud. 2020 Nov 25;6:185. doi: 10.1186/s40814-020-00726-7 (PMC7687695; doi:10.1186/s40814-020-00726-7)
Supplement: Supplementary file 2 — Additional file 2. Stage 1 Consultation. [file 40814_2020_726_MOESM2_ESM.docx]

**Additional File 2: Stage 1 Consultation**

1. Aim

The aim of this consultation was to seek initial qualitative feedback from stakeholders regarding the appropriateness of the proposed BabbleBooster app.

1. Methods

*Participants*

Participants were recruited via a flyer posted on social media. It specifically asked for respondents who could answer any of the three questions positively:

- Did/does your child with autism struggle to learn to produce spoken words and sounds?
- Did/does your child with autism play with smartphone or tablet apps?
- Are you a therapist who works with children with autism who struggle to acquire speech?

Six parents made initial contact with the first author to attend the focus group but for logistical reasons only four attended the group, henceforth referred to as Participants L, E, R and A.

Participant L was the mother of a 7 year-old autistic boy currently educated in a specialist autism unit attached to a mainstream school. She describes him as verbal now, but having had delayed speech and communication development, with his articulation still a bit impacted. Participant E was the mother of an 8 year-old autistic boy currently educated in a specialist school, whom she describes as verbal but cognitively impaired. She describes him as having ongoing articulatory problems. Participant A was the mother of a 10 year-old autistic girl, currently educated in mainstream school with support. She describes her as a very late talker with extreme verbal dyspraxia, which has now resolved. Her current profile is of typical cognitive and verbal ability. Participant R is the mother of an 8 year-old autistic boy currently educated in a language unit. She describes a history of regression and language delay. He is now verbal with remaining language difficulties.

*Procedure*

The focus group was held in March 2017 and was chaired by the first author. Two members of the UCL Computer Science student team who were creating the app were also present.

The focus group took the form of a brief presentation of the proposed app’s features, aims and mechanisms, followed by a series of open ended questions designed to prompt group discussion. Questions are summarised in Table 5. The meeting was audio recorded and transcribed verbatim by the first author. The first author analyzed the data using thematic analysis (Braun & Clarke, 2006) where the content of the focus group transcript was analyzed and themes identified.

Table 5: Focus group discussion guide

| **Theme** | **Questions** |
| --- | --- |
| Technology for young autistic children | *What kind of devices do you/have you in the past let your child play with?*  *Have you encouraged app play? Or placed restrictions on it?*  *When your child was younger did you let them play alone or support their app play?*  *What do you see as the main benefit of the apps your child uses/used?*  *Please share any positive and negative experiences using apps?*  *Do you view the purpose of apps as primarily educational or recreational?*  *Do apps have a limited ‘shelf-life’ with your child if they use them intensively?*  *Which apps have had the most impact on your child?*  *What makes a good app for this population?*  *Which app features are particularly effective?*  *Which app features are particularly annoying?*  *What do you think app-designers need to consider for this population?*  *Any other uses of technology worth mentioning (e.g. Siri, youtube etc)?*  *Have you used any apps that are aimed at improving speech?*  *If so at what point in speech development was your child?*  *Do you think an app could support speech development?*  *What features do you think would be necessary/desirable for such an app?* |
| Feedback on the proposed BabbleBooster app | *Do you think the proposed structure of 5-10 minutes per day, 5 days a week for 12 weeks is acceptable and achievable time commitment?*  *Do you think parents will carry on the visual prompts in day to day?*  *Do you think having the app is likely to create regular practicing routines for families?*  *Would built-in text reminders be a good idea?*  *Parent needs to play app alongside child at all times but should there be a ‘viewing only mode’ for a child who wants to watch video modelling on a loop by themselves?*  *Do you think parents will find it easy enough to rate their child’s speech productions?*  *Is the playback option useful?*  *How important is it for parents to also receive feedback on how the therapy is going?*  *The idea is to take footage of child pre-intervention and determine which sounds are already in the repertoire, and which are not. Any thoughts on this?*  *Should parents have any input into the sound selection process?*  *In your experience were consonants and vowels equally important?*  *How many sounds do you think should be targeted at once?*  *After how long working on a sound in vain should an alternative sound be tried instead?*  *Any comments on how sounds should be modelled?*  *Is cued articulation aspect useful / neutral / distracting for your child?*  *Should there be any choice in the video modelling or option to upload yourself doing it?*  *How could videos be visually improved to make articulation part more salient (e.g. artificially slowed, zooming in, several faces at once, other video effects?)*  *Design will be customisable so that parents can upload reinforcing videos and images - would you use this option? Will this add value for the child?*  *Should there be some ‘placeholder’ videos for those who prefer not to record themselves?*  *Will the child get bored of the reinforcement videos and need them to be changed frequently?*  *Does the reinforcement aspect need to be more dynamic? E.g. you pop bubbles and photos of favourite things are revealed?*  *Would child benefit from watching back their ‘best bits’ at the end of the session? E.g. a clip of a successful production and audio saying – “Yay! [name of child] said ‘b’”*  *Any other issues you see with the app or possible things to improve?*  *Do you think help files might be useful in case of technical problems with the app?* |
| Speech intervention experiences | *How did you try to improve your child’s speech production?*  *Did you feel you understood the developmental sequence of speech production, what skills you should be targeting, and how to do it?*  *What specific therapies did you try for this?*  *Which professionals did you ask for advice on this?*  *Did you agree with focus of therapy on offer?*  *What seemed to be the main barriers to progress with speech?*  *Do you feel it was something that could be helped by repetition or practice or was there a more fundamental difficulty that needed to be addressed first (e.g. attention, low muscle tone, challenging behaviours…)?*  *Was the therapy that you tried able to be adapted to your child’s specific needs/interests? In particular the materials (songs, visuals, videos) or the method of delivery* |

1. Results

Output of the focus group pertaining to the development of the app is summarized below. Due to the limited time (90 minutes), not every question in Table 5 was asked, the discussion flowed in a conversational style.

**Technology:** all parents reported that mobile and tablet devices were inherently motivating for their children, with the most commonly used function being to access to video content online (e.g. via YouTube). Content was often esoteric, user-uploaded and specific to the child’s special interests (e.g. people going on waterslides, opening toys).

**Aim:** all liked the idea of the app and the mirror function. Parents suggested having images that match the sounds would make it more functional. Parents would like to have input on the initial sound selection process.

**Time commitment:** all agreed five minutes per day is an achievable target.

**Cued articulation aspect:** only Participant A had heard of this approach, but when her daughter was minimally verbal she had found it very helpful in progressing speech skills.

**Video modelling aspect:** all agreed this would be a useful feature. Participant R remembered it being hard to get her son to look at her whilst she modelled language, and that is why she thinks he found PECs (a picture exchange communication system) easier that Makaton (a simplified form of sign language, requiring learners to copy manual signs).

**Parent feedback on child productions:** parents unanimously disliked the proposed red “no” button, reporting that their children were very sensitive to ‘getting things wrong’. They suggested changing it to a ‘try again’ button and altering the colours.

**Reinforcing videos:** all agreed that customisable content is a must-have feature of the app. We discussed various ways of supporting parents to create content, for example providing a parent idea sheet or ‘how to’ videos.

1. Discussion

Given the positive feedback regarding aims and features of the app, very few changes were proposed to BabbleBooster. Two pieces of feedback were implemented by the app team. Firstly, rather than just providing the sound and a letter symbol in the sound modelling phase, this was changed to three images corresponding to the sound (e.g. for ‘b’: ‘baby’, ‘ball’, ‘biscuit’). These images were able to be replaced or exchanged by parent customization. Secondly, rather than presenting parents with three choices for feedback buttons (‘yes’, ‘good try’ or ‘no’), this was changed to ‘yes’, ‘good try’ or ‘try again’ and red and green colours were removed, in order to appease parents’ concerns that the red ‘no’ would be interpreted by the child in a negative way.

One suggestion was not possible to incorporate into the intervention, which was the desire for parents to have input into the choice of sound targets. The experimenter systematically selected sound targets from a pre-determined list by following a written protocol (‘Sound Selection protocol, Additional File X). This was done in order to retain experimental control over this aspect of the study and remove potential sources of bias. Nevertheless, it is best practice for speech-language therapists to liaise with those who know the child best when selecting intervention targets, and so for future studies this could be incorporated into the protocol.

A limitation of the focus group was that despite inviting therapists who are involved in language interventions for autistic children, there were none present. This may have been due to the time of day of the focus group, which was primarily scheduled to suit parents. The first author held several bilateral discussions with speech-language and behavioural therapists, to seek additional confirmation that the aims, mechanisms and scope of the intervention were appropriate. No changes to the proposed app arose from these discussions.
