# Supplementary material for: Feasibility of an app-based parent-mediated speech production intervention for minimally verbal autistic children: development and pilot testing of a new intervention
Source: Pilot Feasibility Stud. 2020 Nov 25;6:185. doi: 10.1186/s40814-020-00726-7 (PMC7687695; doi:10.1186/s40814-020-00726-7)
Supplement: Supplementary file 3 — Additional file 3. Stage 2 Consultation. [file 40814_2020_726_MOESM3_ESM.docx]

**Additional File 3: Stage 2 Consultation**

1. Aim

To trial the prototype app to establish whether there were any technical difficulties with it, or if any features needed clarifying or amending prior to the pilot study.

1. Methods

*Participants*

The first author gathered a convenience sample using personal contacts. Participants needed to have access to an android mobile device. Three participants (V, S and J) had children who are or were preverbal with additional needs, one participant (A) had an autistic parent and sibling, three (L, D and E) were typically developing adults. The participants used a range of android devices.

*Procedure*

The first author sent each participant a copy of the app either in an email or via a file sharing service, with a pdf of the proposed instruction booklet, including details of how to download the app. Approximately one week later, the first author sought feedback via telephone and email regarding the ease of download, use, and any technical problems that arose.

1. Results

Feedback from each participant is summarised in Table 6 below.

Table 6: Feedback following app testing exercise

| **Participant** | **Feedback** |
| --- | --- |
| V (mother to R, 18 months old with hemiplegia) | - *the ‘end recording’ button for video capture appears at the side instead of the bottom of the screen - it isn't clear which button to press to end the recording. It would be better either at the bottom centrally or with wording explaining what it was for.* - *R could not attempt any of the sounds, so we were in a loop of attempt -> try again -> attempt -> try again. There were no instructions about having a chance to stop this sound and try another to vary it [the use of the back button here could be made clearer in the instructions.]* - *it would be good if the camera could be activated when you are in the menu to customise photos, i.e. so you don't have to take a photo first and then select it from the gallery* - *one of the audio stimuli was a lot quieter than all the rest (m quieter than a and t)* - *R loved the pics and vids though and found them motivating, just wasn't able to make his own attempts.* |
| S (mother to D, 5 years old with verbal dyspraxia) | - *The app loaded without a problem. The app was easy to use. I went to settings and added some of my own pictures which was surprisingly easy to do. I take it when app is fully developed you would be able to taylor [sic] sounds to those that need to focus on/ choose our own.* - *When I used this with my son (aged 5) he really liked it. He engaged well and copied the cued articulation signs from the video while saying the word. He really liked that it videoed him and watched it back. This will really help as he is working on the shape of his mouth/lips when forming sounds.* - *My son has been having speech therapy for the past three years and we are always trying to find new ways to do things to keep him interested and motivated. This app will certainly do this. I would happily pay for this app.* |
| J (father of an autistic child with dyspraxia) | - *in test mode why are there numbers next to the letters?* - *it would look better if the letters were lower case to show that they are sounds and not capitals* - *I missed the playback on one of the attempts, it's a shame there is no 'replay' button for if the child does a really good attempt* |
| A (has two autistic relatives) | - *I think the starry background should instead be a plain one as it might be distracting* - *some autistic people find mirrors aversive or overly distracting, it would be great to have the option to turn that mirror view off whilst still recording* |
| L (student) | - *Managed to load it fine. On my first few log in attempts it said 'please wait until 0. Remaining: 2' under the password line and the numbers seem to increase and decrease and stop for a while. With a few attempts and refreshes it did let me in though. When I move off the app it logs me off straight away and it takes a few attempts to log back in due to the 'please wait' thingy. Having a go now and all the videos seem to be playing easily and the record function seems to work. Its great!* |
| D (student) | - *I could download the app but not log in – crash report attached* |
| E (retired) | - *managed to load the app but closed itself each time the password was entered (no crash report).* |

1. Discussion

The testing process highlighted technical problems, which were forwarded to the developer to be addressed. Examples of these were: re-recording some of the sound stimuli so that they were all at the same volume, and understanding why the app was failing to log in on some devices.

There were two substantive improvements to the app generated by this process. Firstly, a replay button was added to the test and intervention trial screens, so that the attempt videos can be re-watched by parent-child dyads, providing a further opportunity for feedback and reinforcement. Secondly, an in-app camera function was added, so that parents could take photos of stimuli directly from the customisation menu, in order to facilitate customisation. Other changes were more cosmetic in nature, such as changing capital letters to lower case letters for describing the target sounds, altering the ‘end recording’ button to make it more prominent, removing a ‘busy’ background screen and making it plain. The instruction booklet emphasised how to use the ‘back’ button on android devices, for those only familiar with Apple ones.

One proposed change that could not be made for financial/logistical reasons was the proposal to have the option to turn off the mirror function. This was based on the fact that the participant’s autistic relative has demonstrated an active dislike of mirrors. In hindsight, following the feedback from a couple of participants in the pilot study, this change may have improved the app considerably for the minority of children who did not appear to enjoy the mirror function.

It became apparent in this testing process that not all android devices could support the app reliably. This prompted us to ensure the participants in the pilot study all had access to the same android tablet, which had been tested extensively with the app. Despite this testing process, login problems still persisted in the pilot study, and are considered to be one reason for the variable rates of adherence and engagement observed.
