# Supplementary material for: Feasibility of an app-based parent-mediated speech production intervention for minimally verbal autistic children: development and pilot testing of a new intervention
Source: Pilot Feasibility Stud. 2020 Nov 25;6:185. doi: 10.1186/s40814-020-00726-7 (PMC7687695; doi:10.1186/s40814-020-00726-7)
Supplement: Supplementary file 4 — Additional file 4. Sound Target Protocol. [file 40814_2020_726_MOESM4_ESM.docx]

**Additional File 4: Sound Target Protocol**

This decision tree is designed to generate a list of 9 selected sounds for each child from which target sounds will be drawn. The first three sounds on the list will be the first three targets and from then on, any replacements will go down the list in order.

It is not expected that any child will already be able to produce more than 4 of the sounds mentioned below, therefore each child will have nine sounds on their list.

Decision tree

Stop when 9 unique sounds have been selected

1. Can they say b?

YES - p (if not mastered, otherwise go to 2)

NO - b

2. Can they say n?

YES - go to 3

NO - n

3. Can they say d?

YES - t (if not mastered, otherwise go to 4)

NO - d

4. Can they say ee?

YES - oo (if not mastered, otherwise go to 5)

NO - ee

5. CAn they say a?

YES - go to 6

No - a

6. Can they say p?

YES - m (if not mastered, otherwise go to 7)

No - p

7. Can they say t?

YES - go to 8.

NO - t

8. Can they say oo?

YES - ar (if not mastered, otherwise go to 9)

No - oo

9. Can they say m?

YES - go to 10

NO - m

10. Can they say ar?

YEs - go to 11

No - ar

11. can they say w?

YES - go to 12

No - w

12. Can they say h?

YES - go to 13

NO - h

13. Can they say i?

YES - got to 14

NO - i

14. can they say s?

YES - end

NO - s

This decision process is also presented as a flowchart below:
