# Supplementary material for: Feasibility of an app-based parent-mediated speech production intervention for minimally verbal autistic children: development and pilot testing of a new intervention
Source: Pilot Feasibility Stud. 2020 Nov 25;6:185. doi: 10.1186/s40814-020-00726-7 (PMC7687695; doi:10.1186/s40814-020-00726-7)
Supplement: Supplementary file 5 — Additional file 5. Acceptability Questionnaire. [file 40814_2020_726_MOESM5_ESM.docx]

**Additional File 5: Acceptability Questionnaire**

App name: BabbleBooster (Android Version) Reviewer name: _______________________________ Date: ____________

|  | **4** | **3** | **2** | **1** |
| --- | --- | --- | --- | --- |
| 1. **Relevance:**   **Does the app target the skill it aims to target (=rehearsal of single speech sounds)?** | The app’s focus has a strong connection to the purpose for the app and was appropriate for the learner | The app’s focus is related to the purpose for the app and mostly appropriate for the learner | Limited connection to the purpose for the app and may not be appropriate for the learner | Does not connect to the purpose for the app and not appropriate for the learner |
| 1. **Engagement:**   **Does the app provide enough motivation to hold the interest of the learner?** | Learner is highly motivated to use the app throughout the time | Learner is motivated to use the app most of the time | Learner somewhat engaged, but lost motivation after a short time | Learner avoids the use of the app |
| 1. **Customization:**   **Was the customization able to meet the needs of the learner?** | I was fully able to alter content to meet learner needs | I was able to alter content to meet learner needs but would like further flexibility | I was able to customize app but this was of limited value | Unable to access customization options |
| 1. **In app feedback (reward videos):** | Feedback was clear and strongly aided motivation | Feedback somewhat aided motivation | Feedback was neutral / learner did not connect performance with feedback | Feedback was distracting or counterproductive |
|  | **4** | **3** | **2** | **1** |
| 1. **Layout/Design:** | Layout is simple, logical consistent and easy to navigate with no distracting features | Layout is fairly straightforward with a few minor problems | Layout seemed counterintuitive and unhelpful | I did not like the layout at all |
| 1. **Installation:** | I could launch the app independently from first use | I needed limited initial support in launching the app | I needed frequent support with installation | I was unable to install this app |
| 1. **Navigation:** | Very easy to learn how to use and directions are clear and simple to follow | Easy to learn and direction can be followed | Quite difficult to learn and follow | Very complex to learn – not user-friendly |
| 1. **Support:**   **Evaluate quality of written instructions, video tutorial, in-person training and offline support** | Support was very easy to access, helpful and clear | I found support mostly helpful, clear and accessible | Support was difficult to access and/or of limited use | I did not find the support options useful at all |
| 1. **Reporting:** | I found the progress summary data easy to access, intuitive and encouraging | The progress summary data was quite helpful | The progress output was of limited use | I couldn’t access and/or understand the progress output |
| 1. **Technical performance:**   **Including video capture** | Performs and loads quickly. No issues and very reliable | Performs and Loads quickly. Some minor technical issues. | Loads and performs slowly. Sometimes Crashes. | Crashes fairly often and takes multiple times to open. |

**OVERALL COMMENTS**

1. **What overall rating would you give the app?**
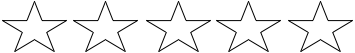

2. **Does this app allow you to do something you were unable to do with your child in the past? If so what?**

**_____________________________________________________________­­_______________________________________________**

**_____________________________________________________________­­_______________________________________________**

**_____________________________________________________________­­_______________________________________________**

1. **What are the strengths of this app?**

**_____________________________________________________________­­_______________________________________________**

**_____________________________________________________________­­_______________________________________________**

**_____________________________________________________________­­_______________________________________________**

1. **What are the weaknesses of this app?**

**_____________________________________________________________­­_______________________________________________**

**_____________________________________________________________­­_______________________________________________**

**_____________________________________________________________­­_______________________________________________**

1. **Comments and recommendations:**

**_____________________________________________________________­­_______________________________________________**

**_____________________________________________________________­­_______________________________________________**
