# Supplementary material for: Feasibility of an app-based parent-mediated speech production intervention for minimally verbal autistic children: development and pilot testing of a new intervention
Source: Pilot Feasibility Stud. 2020 Nov 25;6:185. doi: 10.1186/s40814-020-00726-7 (PMC7687695; doi:10.1186/s40814-020-00726-7)
Supplement: Supplementary file 6 — Additional file 6. Score Breakdown of Acceptability Questionnaire. [file 40814_2020_726_MOESM6_ESM.docx]

**Additional File 6: Score Breakdown of Acceptability Questionnaire**

| Domain | Factor | N | Mean Score | Sd |
| --- | --- | --- | --- | --- |
| 1 | Engagement | 17 | 2.06 | 0.90 |
| 2 | Technical performance | 17 | 2.62 | 0.93 |
| 3 | Reporting | 11 | 2.82 | 1.47 |
| 4 | Customization | 14 | 2.86 | 0.77 |
| 5 | In-app feedback | 16 | 2.97 | 0.87 |
| 6 | Relevance | 17 | 3.35 | 1.06 |
| 7 | Installation | 15 | 3.53 | 0.74 |
| 8 | Navigation | 17 | 3.71 | 0.47 |
| 9 | Layout / design | 17 | 3.82 | 0.39 |
| 10 | Support | 17 | 3.88 | 0.33 |

Note: sd = standard deviation
